# On Ganglion, Simple and Compound

**Published:** 1898-04-16

**Authors:** E. Percy Paton

**Affiliations:** Surgical Registrar to Westminster Hospital, &c


### Medical Progress and Hospital Clinics.

[*The Editor will be glad to receive offers of co-operation and contributions from members of the profession. All letters should be addressed to* The Editor, at the Office, 28 & 29, Southampton Street, Strand, London, W.C.]

### ON GANGLION, SIMPLE AND COMPOUND.

By E. Percy Paton, M.D., M.S., F.R.C.S., Surgical Registrar to Westminster Hospital, &c.

Two varieties of ganglion are usually described, the simple and the compound. The simple ganglion presents itself clinically as a circumscribed, rounded or oval swelling with regular outline, rarely, if ever, larger than a small walnut, and most commonly considerably smaller, and it is attached more or less firmly over a tendon sheath; but since it is attached to the sheath, and not the tendon, it does not move when the latter is put in action. Most commonly it is quite painless, and were it not for the appearance of the swelling no advice would be asked about it; at other times the swelling is said to ache, and the movements of the tendon to the sheath of which it is attached are felt to be somewhat impaired. These swellings are more common in young women than in others, and the obscure pains to which they are occasionally said to give rise are probably mostly neurotic.

Their most common seat is about the tendons on the back of the wrist, and especially the extensor tendons of the thumb; but they may also occur in connexion with other sheaths, such as those about the ankle, dorsum of the foot, and elsewhere.

In many cases no cause for their occurrence can be discovered; but not infrequently considerable and perhaps excessive use of the tendon involved, such as by lawn tennis or violin playing; or, amongst the poor, wringing wet clothes, has immediately preceded the appearance of the swelling, and in some cases its appearance has followed a distinct attack of tenosynovitis.

On dissection the swelling consists of a distinct wall attached to the tendon sheath, and containing a jelly-like translucent material, which exactly resembles glycerine jelly. No connection with the cavity of the sheath to which it is attached can be discovered. In some cases the cyst seems more closely attached to a neighbouring joint than to a tendon sheath, but no connection with the joint cavity is present. Their method of formation is somewhat doubtful; but three theories are described which may not improbably be all true in different cases. The theories are: (1) That the original swelling is merely a hernia of the synovial lining of a sheath through its fibrous covering, which has subsequently become cut off and so forms a cyst; or (2) their formation is due to a cystic degeneration of one of the secreting follicles, which exist as pouch-like crypts in the synovial membrane of the sheath; the least probable theory perhaps is (3) that they are independent new formations, having no connection with a tendon sheath whatever.

Treatment is usually called for, not on account of any pain to which the swelling gives rise, but because of the disfigurement or inconvenience which it causes on account of its size. In some cases the swelling may rupture either spontaneously or by very slight violence, so that a natural cure results; in other cases, however, it may increase to such a size as to considerably impair the movement of the corresponding tendon.

The simplest measure, and one to which no risk or trouble of any sort attaches, is subcutaneous rupture by firm pressure with the two thumbs placed close together over the swelling, or by a smart tap with some solid object, such as the back of a book. By thus rupturing the capsule the contents are extravasated into the surrounding tissues, where, however, they do no harm. Unfortunately the swelling after this treatment is very liable to return when the rent in its capsule has become closed.

Another simple procedure is aseptic puncture, with removal of the contents, followed by pressure. The swelling is carefully washed with soap and water, and then with some antiseptic lotion in common use, such as 1 in 40 carbolic. A clean sharp-pointed tenotomy knife, or Paget's knife, is then taken, and the tumour being fixed with the finger and thumb the knife is thrust through the capsule and moved a little laterally in such a way as that its edge freely incises the capsule. Care must be taken that the capsule is fairly punctured at the first thrust. The swelling is now squeezed before the knife is removed so that the contents come out along its blade, as if this is not done, in some cases in which the capsule has been punctured obliquely, it will be found that it is difficult to get the jelly-like material to commence escaping after the tenotome is removed. When the swelling is empty a pad of antiseptic gauze is placed over the puncture and bandaged firmly on, a short splint being placed on the opposite side of the limb as a counter-pressure, the whole being left on for about a week; not infrequently the result will be a permanent cure, and provided cleanliness has been maintained no possible trouble can arise. Imperfect cleaning of the skin, hands, or tenotome may, however, lead to suppuration, which, as it can speedily track along the tendons, may do considerable damage, as in a case which came to my knowledge recently, in which such extensive suppuration took place as to seriously damage the utility of the arm and hand, and necessitate free incisions all up the arm. Such an untoward result, however, the most ordinary care will prevent.

As an addition to the puncture treatment, the injection of about half a dram of tincture of iodine into the emptied sac is thought by some to make the permanence of the cure more certain.

Short of excision, one other method of treatment may be mentioned, namely, passing an aseptic seton of silk or horsehair through the swelling, and then applying an antiseptic dressing for ten days, after which the seton may be removed.

The only certain method of cure, however, is complete excision. The operation is very simple, and with asepsis the wound is well in a week. The only disadvantage is that it usually requires a general anæsthetic, though it can without much difficulty be done under cocaine by injecting the drug subcutaneously round the swelling. Excision should always be performed should one or other of the methods above described fail to cure.

(*To be continued.*)

